# Activator-Mediated Pyruvate Kinase M2 Activation Contributes to Endotoxin Tolerance by Promoting Mitochondrial Biogenesis

**DOI:** 10.3389/fimmu.2020.595316

**Published:** 2021-01-19

**Authors:** Zhujun Yi, Yilin Wu, Wenfeng Zhang, Tao Wang, Jianping Gong, Yao Cheng, Chunmu Miao

**Affiliations:** Department of Hepatobiliary Surgery, The Second Affiliated Hospital of Chongqing Medical University, Chongqing, China

**Keywords:** sepsis, mitochondrial biogenesis, endotoxin tolerance, pyruvate kinase M2, TEPP-46

## Abstract

Pyruvate kinase M2 (PKM2) is a key glycolysis enzyme, and its effect on macrophages has not been entirely elucidated. Here, we identified that the PKM2 small-molecule agonist TEPP-46 mediated PKM2 activation by inducing the formation of PKM2 tetramer and promoted macrophage endotoxin tolerance. Lipopolysaccharide (LPS)-tolerant mice had higher expression of the PKM2 tetramer, which was associated with a reduced *in vivo* immune response to LPS. Pretreatment of macrophages with TEPP-46 resulted in tolerance to LPS stimulation, as demonstrated by a significant reduction in the production of TNF-α and IL-6. We found that TEPP-46 induced mitochondrial biogenesis in macrophages. Inhibition of mitochondrial biogenesis by mtTFA knockdown effectively inhibited TEPP-46-mediated macrophage tolerance to endotoxins. We discovered that TEPP-46 promoted the expression of PGC-1α and that PGC-1α was the key regulator of mitochondrial biogenesis in macrophages induced by TEPP-46. PGC-1α was negatively regulated by the PI3K/Akt signaling pathway. Knockdown of PKM2 or PGC-1α uniformly inhibited TEPP-46-mediated endotoxin tolerance by inhibiting mitochondrial biogenesis. In addition, TEPP-46 protected mice from lethal endotoxemia and sepsis. Collectively, these findings reveal novel mechanisms for the metabolic control of inflammation and for the induction of endotoxin tolerance by promoting mitochondrial biogenesis. Targeting PKM2 appears to be a new therapeutic option for the treatment of sepsis and other inflammatory diseases.

## Introduction

The systemic inflammatory response and multiple organ failure caused by severe sepsis and septic shock are important causes of high mortality in clinical patients (1, 2). The molecular mechanism is mainly related to the combination of endotoxin and Toll-like receptors (TLRs), which activates the inflammatory pathways of immune cells and then induces the release of a large number of pro-inflammatory factors, such as tumor necrosis factor-α (TNF-α), interleukin 6 (IL-6), and IL-1β (2–4). The release of these proinflammatory cytokines not only induces inflammation but also modulates the immune response (5).

Macrophage endotoxin tolerance or lipopolysaccharide (LPS) tolerance is defined as a hyporesponsive state in response to a secondary lethal dose of LPS following primary low-dose LPS exposure (6, 7). Endotoxin tolerance provides a protective mechanism to reduce the proinflammatory cytokine levels in response to severe infection (8, 9). However, endotoxin tolerance is also a double-edged sword in regulating the immune response (10). For immunocompromised individuals, a prolonged endotoxin-tolerant state allows for the development of secondary infections, increasing mortality from sepsis (11–13). Therefore, understanding the mechanisms controlling endotoxin tolerance is important to design a good time frame for interventions to regulate the immune responses.

Growing evidence suggests that the development of sepsis is closely related to energy metabolism dysfunction in immune cells (14). Activated immune cells, such as macrophages and dendritic cells, also have the ability to switch their energy metabolism from oxidative phosphorylation to glycolysis, which is similar to the “Warburg effect” in tumor cells (15, 16). This switch in energy metabolism is directly involved in the regulation of the inflammatory response (17). Mitochondria are the main organelles of energy metabolism, and mitochondrial dysfunction is also closely related to the development of sepsis (18, 19). Normal mitochondrial function is the basic premise for immune cells to resist the inflammatory response (18). Mitophagy (selective degradation of dysfunctional mitochondria) and mitochondrial biogenesis (generation of new mitochondria) maintain the balance of mitochondrial mass, which plays an important role in maintaining the normal mitochondrial function (19–21). Mitochondrial biogenesis can be induced by cold exposure, oxidative stress, inflammatory cell stress, etc. Under these stimuli, expression of peroxisome proliferator-activated receptor gamma-1 coactivator family (PGC-1α), nuclear respiratory factor 1 (NRF1), NRF2 and mitochondrial transcription factor A (mtTFA/MTFA), which are closely related to mitochondrial biogenesis, was promoted (22, 23). In particular, PGC-1α has been confirmed as an important coordinator that regulates a wide variety of anti-inflammatory and metabolic nuclear genes (22, 24). The AMP-activated protein kinase (AMPK)/sirtuin 1 (SIRT1) pathway regulates mitochondrial biogenesis by inducing PGC-1α (25). However, in some inflammatory diseases, inhibiting the activation of the AMPK/SIRT1 pathway does not inhibit mitochondrial biogenesis, suggesting that there are other ways to regulate mitochondrial biogenesis (25, 26). Overall, inflammation can be modulated by regulating mitochondrial function, but the specific molecular mechanism remains to be further studied.

Pyruvate kinase M2 (PKM2) is a key glycolysis enzyme (27). The enzymatic activity of PKM2 is determined by the configuration of the enzyme into a tetramer, dimer, or monomer (27, 28). The PKM2 tetramer is located in the cytoplasm and is the active form of the enzyme; the PKM2 dimer and monomer are located in the nucleus and play an important role in regulating gene transcription (27, 28). Growing evidence suggests that the transition from a PKM2 tetramer to a PKM2 monomer/dimer (nuclear translocation) plays an important role in promoting the inflammatory response and tumor invasion and proliferation (29–31). LPS induces the formation of the PKM2 monomer/dimer, promotes the transcription of high mobility group box 1 (HMGB1) and NF-κB through interaction with hypoxia-inducible factor 1α (HIF-1α), and further promotes the release of the inflammatory factor IL-1β, which plays an important role in the development of sepsis (32). The small-molecule agonist DASA-58 or TEPP-46 can effectively inhibit the LPS-mediated macrophage inflammatory response by inhibiting the formation of the PKM2 monomer/dimer (33). In addition, TEPP-46 can improve glucose metabolism in podocytes, thus delaying the development of diabetic nephropathy (34). However, it is still unclear what role the PKM2 tetramer plays in inflammatory regulation and what its mechanism is. Overall, these studies have revealed a role for PKM2 in proinflammatory cytokine production and suggest that it is important to regulate PKM2 expression to modulate the inflammatory potential of macrophages.

We report here that the formation of the PKM2 tetramer, triggered by TEPP-46, is a novel mechanism for negatively regulating the inflammatory response and contributes to endotoxin tolerance. TEPP-46-induced activation of PKM2 promotes PGC-1α-mediated mitochondrial biogenesis by inhibiting the PI3K/Akt signaling pathway and promotes endotoxin tolerance by inhibiting the release of the proinflammatory factors TNF-α and IL-6 *in vitro* and *in vivo*. These results not only uncover a novel regulatory mechanism of the inflammatory response by PKM2 but also provide a new therapeutic target to prevent sepsis-mediated immunosuppression.

## Methods and Materials

### Cell Isolation and Culture and Reagents

The isolation methods of peritoneal macrophages (PMs) and Kupffer cells (KCs) from C57BL/6 mice were carried out as previously Hu YC and Li PZ et al. described (35, 36). The RAW264.7 macrophage cell line was purchased from American Type Culture Collection (ATCC). These cell populations were cultured in DMEM (Gibco Life Technologies) with 10% FBS (PAN-Biotechnology) and 1% penicillin–streptomycin (Beyotime Biotechnology) at 37°C, 95% humidity, and 5% CO_2_.

We used antibodies against PKM2 (Cell Signaling, #4053, 1:1,000), PKM1 (Cell Signaling, #7067, 1:1,000), PGC-1α (Cell Signaling, #2178, 1:1,000), PGC-1β (Abcam, ab176328, 1:1,000), p62 (Cell Signaling, #16177, 1:1,000), LC3 (Abcam, ab192890, 1:1,000), mtTFA (Abcam, ab252432, 1:1,000), NRF1 (Abcam, ab221792, 1:1,000), NRF2 (Abcam, ab137550, 1:1,000), p-AMPK (Cell Signaling, #4186, 1:500), AMPK (Cell Signaling, #4150, 1:1,000), SIRT1 (Abcam, ab189494, 1:1,000), p-Akt (Abcam, ab38449, 1:500), Akt (Abcam, ab8805, 1:500), p-PI3K (Cell Signaling, #17366, 1:1,000), PI3K (Cell Signaling, #4255, 1:1,000), and GAPDH (Santa Cruz, sc365062, 1:1,000). LPS (L9641) was purchased from Sigma. The PKM2 activator TEPP-46, Akt activator SC79 and PI3K activator 740 Y-P were from MedChemExpress. Fluorescently labeled secondary antibodies were obtained from ZSGB-BIO. TNF-α and IL-6 enzyme-linked immunosorbent assay (ELISA) kits were from Boster Biological Technology.

### siRNA and Lentivirus Transduction

Small interfering RNA (siRNA)-*PKM2* and control siRNA were prepared by GenePharma (Shanghai), and all short hairpin RNAs (shRNAs) (shRNA-*PGC-1α*, shRNA-*mtTFA* and control shRNAs) were prepared by GeneChem (Shanghai). siRNA and lentiviral transduction were performed according to the manufacturer’s instructions. For siRNA transduction, 2 × 10^5^ RAW264.7 cells (MOI=20) in 2 ml medium with 200 pmol siRNA and 5 µl Lipo8000 (Beyotime Biotechnology). Change the culture medium after 6 h. For lentivirus transduction, 1×10^5^ RAW264.7 cells (MOI=20) in 1 ml medium with 10 μg/ml of polybrene (GeneChem) were incubated with 2 µl lentivirus. After 72 h of culture, western blot were used to analyze the transduction efficiency. The siRNA-*PKM2* sequences refer to Goldberg’s work (siRNA 27 in Goldberg’s work) (37). The sequences of siRNAs were as follows: mouse PKM2-siRNA (5’-AGGCAGAGGCUGCCAUCUA-3’) and control siRNA (5’-UUCUCCGAACGUGUCACGU-3’). The target sequences of shRNAs were as follows: mouse PGC-1α-shRNA (5’-CCGGCCAGAACAAGAACAACGGTTTCTCGAGAAACCGTTGTTCTTGTTCTGGTTTTTG-3’), mouse mtTFA–shRNA (5’-CCGGCGGAGACATCTCTGAGCATTACTCGAGTAATGCTCAGAGATGTCTCCGTTTTTG-3’) and control shRNA (5’-TTCTCCGAACGTGTCACGT-3’).

### Endotoxin Tolerance and Acute Endotoxemia and Sepsis Mouse Models

Male C57BL/6 mice (6–8 weeks old, 22–25 g) were purchased from the Experimental Animal Center of Chongqing Medical University and were maintained under the guidelines of the Animal Care and Use Committee of Chongqing Medical University. Animals were fed standard rodent chow in a temperature-controlled environment with 50% humidity and 12 h light/dark cycles in a cage of five mice. To develop the endotoxin tolerance mouse model, C57BL/6 mice were preinjected with a low dose of LPS (8 μg/kg body weight, peritoneally) for 16 h and then challenged with a lethal dose of LPS (8 mg/kg, peritoneally). Survival was monitored every hour for the next 10 h. An endotoxemia mouse model was induced in male C57BL/6 mice by LPS and a sepsis mouse model was induced by cecal ligation and puncture (CLP). For LPS-induced endotomexia, mice preinjected with saline solution or TEPP-46 (50 mg/kg, intraperitoneally) for 4 h were restimulated with LPS (5 mg/kg, intraperitoneally). Sepsis was induced in mice by CLP as previously Gong W et al. described (38). In brief, the mice were anesthetized with 2% isoflurane inhalation, and iodophor was used to disinfect the abdomen. Then, a 2 cm midline incision was made to expose the cecum and ligate the distal cecal tip with 4-0 silk. The cecum was punctured twice with a 22-gauge needle, and a small amount of feces was extruded. Then, the cecum was returned to the abdominal cavity, and the wound was closed. Sham mice underwent only laparotomy. Survival of these mice was monitored every day for the next week. The present study was approved by The Research Ethics Committee of Chongqing Medical University (No. 2017-36).

### ELISA

The levels of TNF-α and IL-6 in mouse serum and cell supernatant were determined by using an ELISA kit from Boster Biological Technology according to the manufacturer’s instructions.

### Cell Viability of PMs and RAW264.7 Cells

Cell viability was used to evaluate the cytotoxicity of TEPP-46 to PMs and RAW264.7 cells. In brief, 5 × 10^3^ cells were seeded in each well of a 96-well plate in 100 μl of medium and cultured for 24 h. Then, 10 μl of Cell Counting Kit-8 (CCK8, C0037, Beyotime Biotechnology) was added to each well, and the cells were incubated at 37°C for 1 h. The absorbance of each well was detected at 450 nm using a microplate reader.

### Western Blot

Western blot analysis was carried out as previously Palsson-McDermott EM and Hu YC et al. described (33, 35). It is worth noting that the Western blot experiment performed for PKM2 was different from the Western blot experiment performed for other proteins. Due to the need to detect PKM2 protein expression with different configurations, the total protein extracted from the cells cannot be heat denatured. In addition, nondenatured gel sample loading buffer (Beyotime Biotechnology, P0016) replaced the SDS-PAGE sample loading buffer (Beyotime Biotechnology, P0015), and native PAGE electrophoresis buffer (Beyotime Biotechnology, P0014F) replaced the SDS-PAGE electrophoresis buffer (Beyotime Biotechnology, P0014A) in Western blot experiments of PKM2 but not in the Western blot experiments of other proteins. The other experimental steps were the same as a routine Western blot experiment. In brief, cells were harvested and lysed with a whole-protein extraction kit (KeyGen). Protein concentration was detected by a BCA protein assay kit (Beyotime Biotechnology). Except for the protein used to detect PKM2, the other proteins were mixed with loading buffer and boiled in water for 10 min. In total, 10~20 μg protein lysate was separated by SDS−PAGE with 8%~12% gels and transferred to PVDF membranes (Millipore), which were blocked with 5% BSA for 1 h at room temperature. The membranes were incubated with antibodies overnight at 4°C. The target bands were detected by using the corresponding secondary antibodies. Bands were analyzed using Quantity One software (Bio−Rad) after incubation with enhanced chemiluminescence reagent (MedChemExpress) at room temperature for 2~10 s.

### Detection of PKM2 Nuclear Translocation by Immunofluorescence Staining

After KCs successfully established tolerant and nontolerant models, the cells were fixed with 4% paraformaldehyde for 10 min. After a brief wash with PBS, the cell membrane was lysed with 0.1% Triton for 10 min. Then, the membrane was blocked with goat serum for 1 h. The cells were incubated with a rabbit mAb anti PKM2 (Cell Signaling, #4053, 1:100) overnight at 4°C. The next day, the cells were incubated with the corresponding fluorescent secondary antibody (ZSGB-BIO) for 1 h at room temperature. An appropriate amount of 4’-6-Diamidino-2-phenylindole (DAPI, MedChemExpress) was added to each plate for 3 min, and the protein expression of PKM2 was observed under an inverted microscope.

### Real-Time Reverse Transcription-Polymerase Chain Reaction Analysis of Gene Expression

The RT-PCR experiment was used to detect the M2 polarization of RAW264.7 cells. The RT-PCR experimental procedure was performed as previously Hu YC et al. described (35). In brief, total RNA was extracted from RAW264.7 cells using TRIzol^®^ reagent (Invitrogen), and then the RNA was reverse-transcribed to cDNA with the PrimeScript™ RT Reagent Kit (TaKaRa Biotechnology). Primers for RT-PCR were obtained from Sangon Biotech. The expression of the target gene was normalized to that of GAPDH. The primers used were as follows: Arg1, forward 5’-CACTACCCCACCCCACTC-3’ and reverse 5’-AACGGAGCAAGACCCTGT-3’; CD206, forward 5’-GAAGCCAAGGTCCAGAAA-3’ and reverse 5’-TGTTGAAAGCGTATGTCCA-3’; and GAPDH, forward 5’-CCTTCCGTGTCCCCACT-3’ and reverse 5’-GCCTGCTTCACCACCTTC-3’. The relative gene expression was analyzed using the 2^−ΔΔCq^ method (39).

### mtDNA Copy Number Detection

Total DNA was extracted from cells using the DNeasy Blood & Tissue Kit (Qiagen) and used for the detection of mtDNA copy number by RT-PCR using SYBR Green (TaKaRa Biotechnology) and an ABI PRISM 7900 Sequence Detection system (Thermo Fisher Scientific). The relative mtDNA copy number was determined by comparing the level of the mitochondrial NADH dehydrogenase subunit 1 (MTND1) gene (primers, forward 5’-GAGAACAAAGGTGAGAAGCAA-3’ and reverse 5’-TCCACACAGATCCAGCATAA-3’) to that of the nuclear reference gene B2M (primers, forward 5’-AGCAGAGAATGGAAAGTCAAA-3’ and reverse 5’-GATGGATGAAACCCAGACA-3’). The relative mtDNA copy number was defined as the total amount of mtDNA divided by the total amount of nuclear DNA.

### Mitochondrial Mass Detection

The detection of mitochondrial mass was the same as that described in a previous study (40). In brief, the uptake of 2.5 μM nonyl acridine orange (NAO, a dye that localizes to cardiolipin on the inner mitochondrial membrane) by PMs/KCs/RAW264.7 cells over 30 min was determined by measuring fluorescence by using a flow cytometer.

### Autophagy Detection

Western blot analysis of the LC3-II and p62 protein levels was used to assess the level of autophagy in RAW264.7 cells stimulated with TEPP-46 for 2 h.

### Mitochondrial Respiration Detection

RAW264.7 cells with or without TEPP-46 treated in medium at pH 7.4 were transferred to the wells of an XF96 Seahorse assay plate to determine the mitochondrial oxygen consumption rate (OCR) during mitochondrial stress test by using the Seahorse XF96^e^ Extracellular Flux analyzer as previously John D et al. described (40). Before start the experiment, the cells were incubated at 37°C without CO_2_ for 1 h. The OCR was measured at the baseline and following the sequential addition of 1 μM oligomycin to inhibit ATP synthase, and 0.5 μM carbonyl cyanide 4-(trifluoromethoxy) phenylhydrazone (FCCP) was added to yield maximal uncoupled respiration. Non-mitochondrial respiration was determined by adding 1 μM rotenone plus 1 μM antimycin A. Next, we quantitative analysis of mitochondrial basal respiration, ATP-linked respiration and maximal respiratory of the RAW264.7 cells with or without TEPP-46 treat.

### Statistical Analysis

Statistical analyses were performed using SPSS 17.0 software (SPSS, Inc.). Data are presented as the mean ± SD of ≥3 independent biological replicates. Student’s *t* test was performed for the comparison of parameters between two groups. One−way ANOVA and Tukey’s test were performed to compare multiple groups. A *p*-value less than 0.05 was considered significant.

## Results

### PKM2 Activation Promotes Macrophage Endotoxin Tolerance

**Figure 1:**
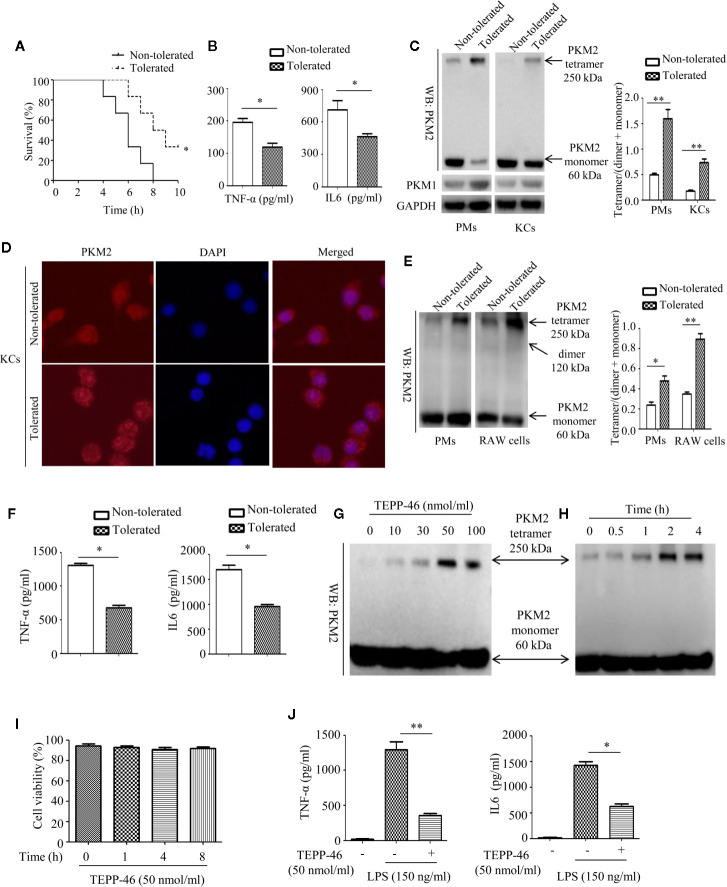
Endotoxin tolerance promotes PKM2 tetramer formation of macrophages *in vivo* and *in vitro*. **(A)** Mouse survival was monitored every hour after constructing nontolerant and tolerant models (*n*=6 per group). **(B)** Serum TNF-α and IL-6 levels from tolerant and nontolerant mice were measured using ELISA (*n*=3). **(C)** The protein expression of PKM2 and PKM1 in PMs and KCs isolated from tolerant and nontolerant mice was measured using Western blot (*n*=3). **(D)** The protein expression of PKM2 (red) and DAPI staining for the nucleus (blue) in KCs isolated from tolerant and nontolerant mice were measured using immunofluorescence. **(E)** Protein expression of PKM2 in tolerant and nontolerant PMs and RAW264.7 cells *in vitro* (*n*=3). **(F)** Supernatant TNF-α and IL-6 levels in RAW264.7 cells were measured using ELISA (*n*=3). **(G, H)** Protein expression of PKM2 in RAW264.7 cells after stimulation with TEPP-46 at different concentrations and for different times (*n*=3). **(I)** Cell viability of RAW264.7 cells stimulated with 50 nmol/ml TEPP-46 for different times was measured using CCK8 (*n*=3). **(J)** Supernatant TNF-α and IL-6 levels in RAW264.7 cells after treatment with TEPP-46 for 2 h and LPS for 24 h (*n*=3). *p < 0.05, **p < 0.01.

The enzymatic activity of PKM2 is, in part, determined by the configuration of the enzyme into a tetramer, dimer, or monomer (27, 28). The PKM2 tetramer is located in the cytoplasm and is the active form of enzyme; the PKM2 dimer and monomer are located in the nucleus and play an important role in regulating gene transcription (27, 28). To determine whether PKM2 expression can be modulated by LPS exposure during the establishment of endotoxin tolerance, we intraperitoneally preinjected mice with saline (nontolerant) or a low dose of LPS (8 μg/kg body weight, tolerant) for 16 h, challenged all the mice with a lethal dose of LPS (8 mg/kg), and then monitored the survival of the mice. As predicted, exposure to a low dose of LPS prior to the lethal dose of LPS increased survival and decreased the serum levels of the inflammatory cytokines TNF-α and IL-6 in mice (**Figures 1A, B**). Then, we isolated PMs and liver macrophages (KCs) from tolerant and nontolerant mice (4 h after a lethal dose of LPS) and evaluated the protein expression of PKM2 in the cells. Interestingly, we found a higher protein level of the PKM2 tetramer and a lower protein level of the PKM2 dimer/monomer in both the PMs and KCs of tolerant mice compared to those of nontolerant mice, suggesting that endotoxin tolerance inhibits the nuclear translocation of PKM2 (**Figure 1C**). The protein level of PKM1, a member of the pyruvate kinase family, did not change significantly (**Figure 1C**). Immunofluorescence further confirmed that exposure to a low dose of LPS prior to the lethal dose of LPS inhibited the nuclear translocation of PKM2 in PMs (data not shown) and in KCs (**Figure 1D**). Next, we performed an *in vitro* experiment by pre-stimulating PMs isolated from healthy mice or RAW264.7 macrophages with a low dose of LPS (10 ng/ml, tolerant) or PBS (nontolerant) for 24 h and then restimulating the cells with a high dose of LPS (150 ng/ml) for 24 h. Consistent with *in vivo* experiments, compared to nontolerant cells, tolerant cells had a significantly increased protein level of the PKM2 tetramer and significantly decreased expression of the PKM2 dimer/monomer (**Figure 1E**). The supernatant levels of TNF-α and IL-6 in also significantly decreased in tolerant cells (**Figure 1F**). These *in vivo* and *in vitro* studies confirmed that macrophage tolerance to endotoxins inhibits the nuclear translocation of PKM2 induced by LPS and promotes the formation of the PKM2 tetramer.

TEPP-46 is a small molecular agonist of PKM2 and can inhibit the nuclear translocation of PKM2 and promote the formation of a PKM2 tetramer (41). As predicted, stimulation of RAW264.7 cells with different concentrations of TEPP-46 (0–100 nmol/ml) for 2 h or 50 nmol/ml TEPP-46 for different times (0–4 h) dose- and time-dependently promoted the protein level of the PKM2 tetramer (**Figures 1G, H**). We also assessed whether TEPP-46 has cytotoxic effects. We found that 50 nmol/ml TEPP-46 had no significant effect on cell viability under different stimulation times (0–8 h) (**Figure 1I**). To further investigate the role of the PKM2 tetramer (activated configuration of PKM2) in the establishment of endotoxin tolerance, we pretreated RAW264.7 cells with either PBS or TEPP-46 (50 nmol/ml) for 2 h and then restimulated these cells with LPS (150 ng/ml) for 24 h. We found that compared to cells treated with only LPS, RAW264.7 cells pretreated with TEPP-46 produced levels of TNF-α and IL-6 that were significantly decreased by 76% and 58%, respectively, in the culture supernatants (**Figure 1J**). Collectively, these results confirm the ability of TEPP-46 to induce endotoxin tolerance in macrophages by promoting the formation of the PKM2 tetramer.

### Mitochondrial Biogenesis Is Induced in PKM2-Activated Macrophages

**Figure 2:**
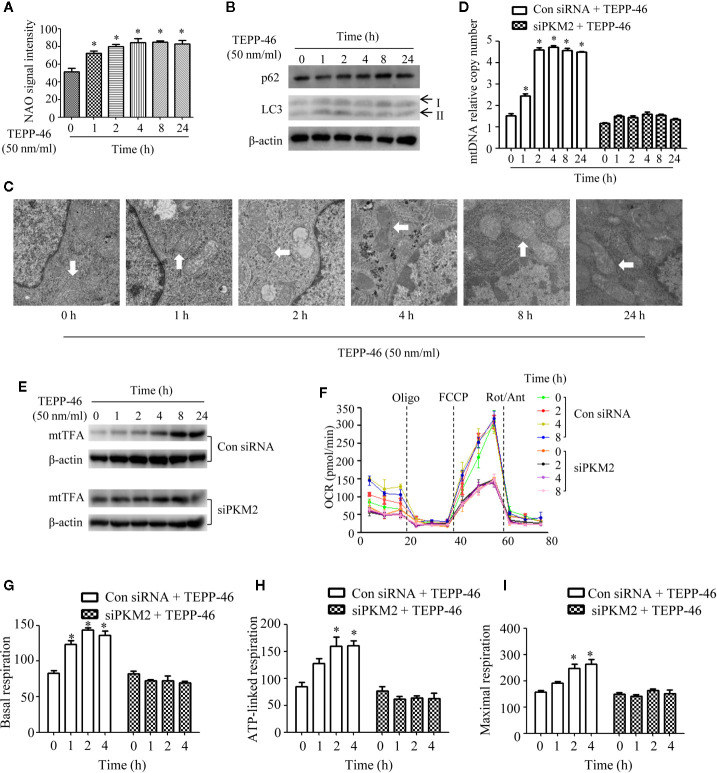
Mitochondrial biogenesis is induced in PKM2-activated macrophages. **(A)** Mitochondrial mass was assessed by measuring the uptake of NAO by using flow cytometry (*n*=3). **(B)** The autophagy level of RAW264.7 cells after stimulation with TEPP-46 was assessed by measuring the protein expression of p62 and LC3 using Western blotting (*n*=3). **(C)** The microstructure of RAW264.7 cells stimulated with TEPP-46 was measured using transmission electron microscopy (TEM) (*n*=3). **(D)** mtDNA copy number was determined by measuring MTND1 relative to B2M using RT-PCR in RAW264.7 cells transducted with a control siRNA (con siRNA) or siPKM2 (*n*=4). **(E)** Western blot analysis of mtTFA in control or PKM2 knockdown RAW264.7 cells were stimulated with TEPP-46 for different times (*n*=3). **(F)** The mitochondrial OCR was measured in control or PKM2 knockdown RAW264.7 cells were stimulated with TEPP-46 by using a Seahorse XF96e Extracellular Flux analyzer. Dashed vertical lines indicate the addition of 1 μM oligomycin (Oligo), 0.5 μM carbonyl cyanide 4-(trifluoromethoxy) phenylhydrazone (FCCP), and 1 μM rotenone plus 1 μM antimycin A (Rot/Ant) (*n*=3). **(G–I)** Quantitative analysis of basal respiration, ATP-linked respiration and maximal respiration in control or PKM2 knockdown RAW264.7 cells were stimulated with TEPP-46 for different times (*n*=3). *p < 0.05.

Mitophagy and mitochondrial biogenesis maintain the relative balance of mitochondrial mass, which plays an important role in maintaining the normal function of cells (19–21). A study showed that PKM2 activation may inhibit mitochondrial dysfunction of podocytes induced by high glucose (34). However, the effect of PKM2 activation on the mitochondrial function of macrophages is still unclear. Thus, we assessed the mitochondrial function of macrophages stimulated with TEPP-46. We observed a significantly increased level of the internal membrane marker cardiolipin in RAW264.7 cells stimulated with TEPP-46 (50 nmol/ml) for 0–24 h, which indicates an increase in mitochondrial mass under stimulation with TEPP-46 (**Figure 2A**). We next studied whether the increase in mitochondrial mass after treatment with TEPP-46 was caused by inhibiting the degradation of mitochondria (mitophagy). However, we did not observe changes in the protein levels of the autophagy markers LC3-II and p62 in RAW264.7 cells stimulated with TEPP-46 (50 nmol/ml) for 0–24 h (**Figure 2B**). In addition, there was no obvious change in mitochondrial morphology and no obvious decrease in the number of lysosomes stimulated with TEPP-46 (**Figure 2C**). The above results indicate that the overall autophagy level does not change under TEPP-46 stimulation. This means that the activation of PKM2 by TEPP-46 increased the level of mitochondrial mass but did not change the level of mitophagy in RAW264.7 cells. This reflects that mitochondrial mass may be increased by activation of mitochondrial biogenesis. As predicted, we observed a significant increase in mitochondrial DNA (mtDNA) copy number in RAW264.7 cells stimulated with TEPP-46 (50 ng/ml) for 0–24 h, and these results were consistent with the increased protein level of mitochondrial transcription factor A (mtTFA), a key regulator of mitochondrial biogenesis bound to mtDNA (**Figures 2D, E**). We repeated the above experiments after silencing the *PKM2* gene in RAW264.7 cells with siRNA-PKM2 (siPKM2). We found that TEPP-46 failed to increase the mtDNA copy number and the protein level of mtTFA in *PKM2*-knockdown RAW264.7 cells, suggesting that the effect of TEPP-46 is dependent on PKM2 (**Figures 2D, E**).

The effect of TEPP-46 on mitochondrial function was further assessed by measuring the mitochondrial oxygen consumption rate (OCR). We found that the basal respiration, ATP-linked respiration and maximal respiration were significantly increased in TEPP-46-treated RAW264.7 cells (**Figures 2F–I**). This effect of TEPP-46 was eliminated in *PKM2*-knockdown cells (**Figures 2F–I**). This indicates that the activation of PKM2 induced by TEPP-46 enhanced the mitochondrial respiratory capacity of macrophages. Collectively, we observed that PKM2 activation induced by TEPP-46 enhanced mitochondrial function by promoting mitochondrial biogenesis and increasing the mitochondrial mass but did not change the level of mitophagy.

### Inhibiting Mitochondrial Biogenesis Reverses PKM2 Activation-Induced Macrophage Endotoxin Tolerance

**Figure 3:**
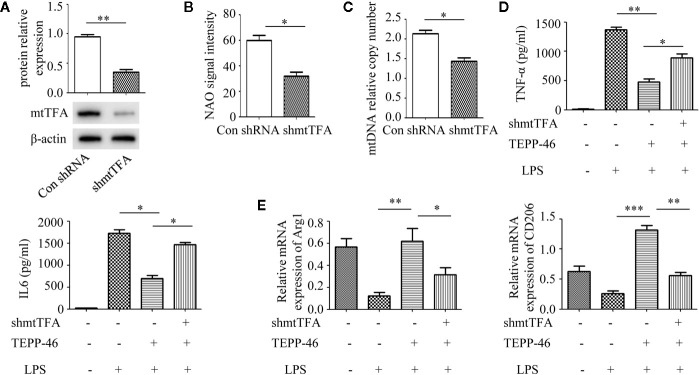
Mitochondrial biogenesis is required for PKM2 activation-induced endotoxin tolerance. **(A)** RAW264.7 cells were treated with control shRNA or shmtTFA lentiviruses for 72 h. Protein expression of mtTFA was measured by Western blot (*n*=3). **(B)** NAO signal intensity was used to assess mitochondrial mass in control RAW264.7 cells (con shRNA) and in *mtTFA* knockdown RAW264.7 cells (shmtTFA) (*n*=4). **(C)** mtDNA copy number was determined by measuring MTND1 relative to B2M using RT-PCR (*n*=3). **(D)** Supernatant TNF-α and IL-6 levels in RAW264.7 cells stimulated with LPS and/or TEPP-46 were measured using ELISA (*n*=3). **(E)** RAW264.7 polarization level was assessed by measuring the relative mRNA expression of *Arg1* and *CD206* to that of GAPDH using RT-PCR (*n*=3). *p < 0.05, **p < 0.01, ***p < 0.001.

Although we demonstrated that PKM2 activation by TEPP-46 contributes to endotoxin tolerance and that PKM2 activation also induces mitochondrial biogenesis in macrophages, we do not know whether PKM2 activation promotes endotoxin tolerance by inducing mitochondrial biogenesis. mtTFA is a key regulator of mitochondrial biogenesis bound to mtDNA (42). Several lines of evidence have suggested that inhibiting the expression of mtTFA could effectively inhibit mitochondrial biogenesis in various cells (42, 43). The mtTFA gene was knocked down in RAW264.7 cells by shRNA-mtTFA (shmtTFA) lentiviruses transduction. As predicted, the protein level of mtTFA decreased significantly after lentiviral transduction for 72 h (**Figure 3A**). Next, we observed a significant decrease in mitochondrial mass and mtDNA copy number in *mtTFA* knockdown RAW264.7 cells, suggesting that silencing *mtTFA* in RAW264.7 cells inhibits mitochondrial biogenesis (**Figures 3B, C**). We further assessed the effect of silencing *mtTFA* on endotoxin tolerance induced by PKM2 activation in macrophages. We found that silencing *mtTFA* inhibited the endotoxin tolerance of RAW264.7 cells induced by PKM2 activation, which was reflected in the levels of the inflammatory cytokines TNF-α and IL-6 in the supernatant of the *mtTFA* knockdown group (shmtTFA + TEPP-46 + LPS), which increased by 85% and 111%, respectively, compared to the levels in the endotoxin tolerance group (TEPP-46 + LPS) (**Figure 3D**). Endotoxin tolerance can inhibit LPS-mediated M1 type polarization of macrophages and promote the expression of M2 type markers, such as Arg1 and CD206 (44, 45). In this study, we found that LPS inhibited expression of Arg1 and CD206, and pretreatment with TEPP-46 promoted expression of Arg1 and CD206, suggesting that pretreatment with TEPP-46 promoted M2 polarization of macrophages (**Figure 3E**). Furthermore, TEPP-46 failed to promote the expression of Arg1 and CD206 after silencing of *mtTFA*, suggesting that the effect of TEPP-46 is dependent on mtTFA (mitochondrial biogenesis) (**Figure 3E**). Overall, these data indicate that inhibiting mitochondrial biogenesis can effectively inhibit the macrophage tolerance to endotoxins induced by PKM2 activation.

### PGC-1α Is the Key Regulator of PKM2 Tetramer-Induced Mitochondrial Biogenesis

**Figure 4:**
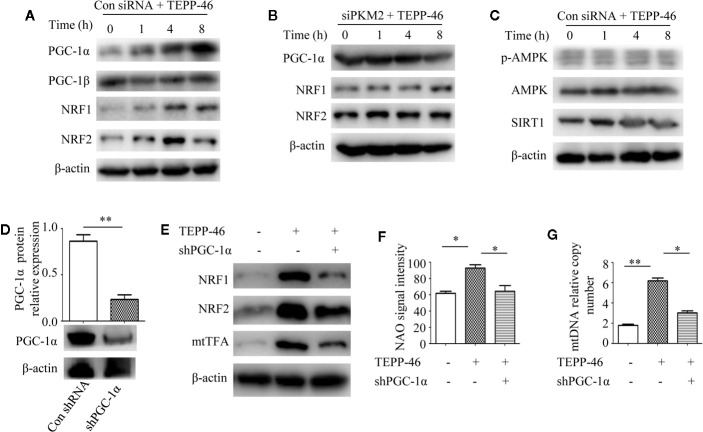
PGC-1α is required for PKM2 activation-induced mitochondrial biogenesis. **(A)** Western blot analysis of PGC-1α, PGC-1β, NRF1, and NRF2 in RAW264.7 cells transduct with control siRNA after stimulation with TEPP-46 for 0–8 h (*n*=3). **(B)** Western blot analysis of PGC-1α, NRF1, and NRF2 in *PKM2*-knockdown RAW264.7 cells after stimulation with TEPP-46 for 0-8 h. (*n*=3). **(C)** Western blot analysis of p-AMPK, AMPK and SIRT1 in RAW264.7 cells transduct with control siRNA after stimulation with TEPP-46 for 0-8 h. (*n*=3). **(D)** RAW264.7 cells were treated with control shRNA or shPGC-1α lentiviruses for 72 h. Protein expression of PGC-1α was measured by Western blot (*n*=3). **(E)** Western blot analysis of NRF1, NRF2 and mtTFA in RAW264.7 cells. (*n*=3). **(F)** NAO signal intensity was used to assess mitochondrial mass in control RAW264.7 cells and in *PGC-1α* knockdown RAW264.7 cells (shPGC-1α) after stimulation with TEPP-46 (*n*=3). **(G)** mtDNA copy number was determined by measuring MTND1 relative to B2M using RT-PCR (*n*=3). *p < 0.05, **p < 0.01.

PGC-1α, NRF1, NRF2, and mtTFA are the key regulatory factors that promote mitochondrial biogenesis (22, 23). The above results have shown that PKM2 activation induced by TEPP-46 could promote the expression of mtTFA (**Figure 2E**). Thus, we further assessed the effect of PKM2 activation on the expression of PGC-1α, NRF1 and NRF2. As predicted, the protein levels of PGC-1α, NRF1, and NRF2 increased in a time-dependent manner under stimulation with TEPP-46 (50 nmol/ml) (**Figure 4A**). The protein level of PGC-1β was not significantly changed (**Figure 4A**). Furthermore, we found that TEPP-46 failed to increase the protein levels of PGC-1α, NRF1 and NRF2 in *PKM2-*knockdown RAW264.7 cells, suggesting that the effect of TEPP-46 is dependent on PKM2 (**Figure 4B**). These results indicate that TEPP-46 promotes mitochondrial biogenesis by inducing PKM2 activation and by further promoting the expression of PGC-1α, NRF1, NRF2, and mtTFA. The AMP-activated protein kinase (AMPK)/sirtuin 1 (SIRT1)/PGC-1α pathway is a classical pathway that induces mitochondrial biogenesis (46). Therefore, we asked whether pretreatment with TEPP-46 could activate the AMPK/SIRT1/PGC-1α signaling pathway. However, we did not observe significant changes in the protein levels of p-AMPK, AMPK, and SIRT1, suggesting that TEPP-46-promoted expression of PGC-1α was not dependent on activation of the AMPK/SIRT1 pathway (**Figure 4C**). To further confirm that TEPP-46 induces mitochondrial biogenesis by activating PGC-1α, we silenced the *PGC-1α* gene in RAW264.7 cells by lentiviral transduction (**Figure 4D**). We found that silencing *PGC-1α* inhibited TEPP-46-induced expression of NRF1, NRF2 and mtTFA (**Figure 4E**). Furthermore, both the mitochondrial mass and mtDNA copy number were decreased in *PGC-1α*-knockdown cells compared to in TEPP-46-treated cells (**Figures 4F, G**). These data collectively suggest that PGC-1α is the key regulator of mitochondrial biogenesis induced by TEPP-46-mediated activation of PKM2.

### PKM2 Tetramer Activates PGC-1α by Inhibiting the PI3K/Akt Signaling Pathway

**Figure 5:**
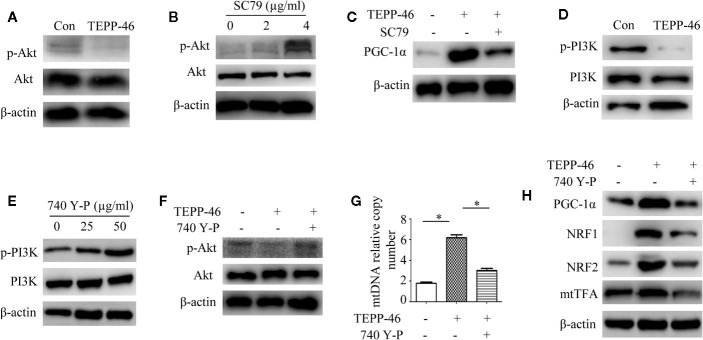
The PI3K/Akt signaling pathway is required for PKM2 activation-induced PGC-1α expression. **(A)** Western blot analysis of p-Akt in normal RAW264.7 cells (Con) and TEPP-46-treated cells (TEPP-46) (*n*=3). **(B)** Western blot analysis of p-Akt in RAW264.7 cells after stimulation with different concentrations of SC79 for 30 min (*n*=3). **(C)** Western blot analysis of PGC-1α in RAW264.7 cells after stimulation with TEPP-46 and/or SC79 (*n*=3). **(D)** Western blot analysis of p-PI3K in RAW264.7 cells stimulated with TEPP-46 (*n*=3). **(E)** Western blot analysis of p-PI3K in RAW264.7 cells after stimulation with different concentrations of 740 Y-P for 24 h (*n*=3). **(F)** Western blot analysis of p-Akt in RAW264.7 cells after stimulation with TEPP-46 and/or 740 Y-P (*n*=3). **(G)** mtDNA copy number was determined by measuring MTND1 relative to B2M using RT-PCR (*n*=3). **(H)** Western blot analysis of PGC-1α, NRF1, NRF2 and mtTFA in RAW264.7 cells after stimulation with TEPP-46 and/or 740 Y-P (*n*=3). *p < 0.05.

We have confirmed that PKM2 activation induced by TEPP-46 promotes the activation of PGC-1α but not by activating the AMPK/SIRT1 signaling pathway. This means that activated PKM2 may promote PGC-1α by activating other pathways. Since Akt has been shown to be a negative regulator of PGC-1α (47), we then asked whether Akt participates in the TEPP-46-induced regulation of PGC-1α. We found that the protein level of phosphorylated Akt (p-Akt) was significantly decreased in RAW264.7 cells stimulated by TEPP-46 (50 nmol/ml) for 2 h (**Figure 5A**). SC79 is a small-molecule agonist of Akt, which promotes phosphorylation of Akt (**Figure 5B**) (48). RAW264.7 cells were pretreated with SC79 (4 µg/ml) for 30 min and then restimulated with TEPP-46 (50 nmol/ml) for 2 h. We found that pretreatment with SC79 effectively inhibited the protein level of PGC-1α induced by TEPP-46 (**Figure 5C**). These results indicate that TEPP-46-induced activation of PKM2 regulates PGC-1α by inhibiting Akt phosphorylation.

Phosphatidylinositol-3-kinase (PI3K) is an intracellular phosphatidylinositol kinase that promotes Akt activation (49). The PI3K/Akt signaling pathway plays an important role in the regulation of tumors and inflammation (50, 51). Since inducing the formation of the PKM2 monomer/dimer could promote the invasion and migration of tumor cells by activating the PI3K/Akt signaling pathway (52), we then asked whether the PKM2 tetramer induced by TEPP-46 could regulate Akt through PI3K in macrophages. We found that the expression of phosphorylated PI3K (p-PI3K) was significantly decreased in RAW264.7 cells stimulated by TEPP-46 (**Figure 5D**). 740 Y-P is a target agonist of PI3K, which promotes phosphorylation of PI3K (**Figure 5E**) (53). RAW264.7 cells were pretreated with 740 Y-P (50 µg/ml) for 24 h and then restimulated with TEPP-46 (50 nmol/ml) for 2 h. We found that the inhibitory effect of TEPP-46 on p-Akt was blocked by 740 Y-P (**Figure 5F**). Furthermore, pretreatment with 740 Y-P inhibited the mtDNA copy number and the protein levels of PGC-1α, NRF1, NRF2 and mtTFA induced by TEPP-46 (**Figures 5G, H**). Collectively, these data indicate that TEPP-46-induced activation of PKM2 promotes PGC-1α expression by inhibiting the PI3K/Akt signaling pathway.

### Knockdown of PGC-1α Inhibits PKM2 Activation-Mediated Endotoxin Tolerance

**Figure 6:**
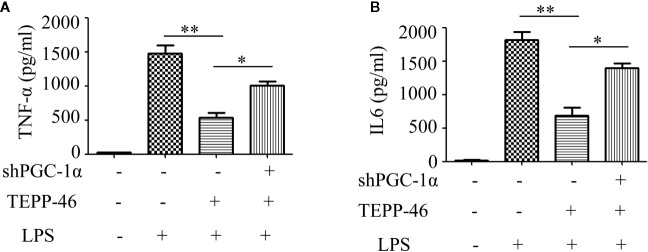
PGC-1α is required for PKM2 activation-induced endotoxin tolerance. **(A, B)** Supernatant TNF-α and IL-6 levels in control RAW264.7 cells or *PGC-1α* knockdown RAW264.7 cells stimulated with LPS and/or TEPP-46 were measured using ELISA (*n*=3). *p < 0.05, **p < 0.01.

We have confirmed that PGC-1α is a key regulator of mitochondrial biogenesis induced by activated PKM2. Next, we assessed the effect of PGC-1α on endotoxin tolerance mediated by PKM2 activation. TEPP-46-induced PKM2 activation promotes macrophage tolerance to endotoxins (**Figure 1J**). We found that knockdown of the *PGC-1α* gene inhibited TEPP-46-mediated endotoxin tolerance. This was evidenced by the levels of TNF-α and IL-6 in the supernatants of *PGC-1α*-knockdown cells (shPGC-1α + TEPP-46 + LPS) significantly increasing by 86% and 102%, respectively, compared to those of cells without *PGC-1α* gene knockdown (TEPP-46 + LPS) (**Figures 6A, B**). This indicates that knockdown of *PGC-1α* inhibits PKM2 activation-mediated endotoxin tolerance.

### TEPP-46 Protects Mice From Endotoxemia and Sepsis

**Figure 7:**
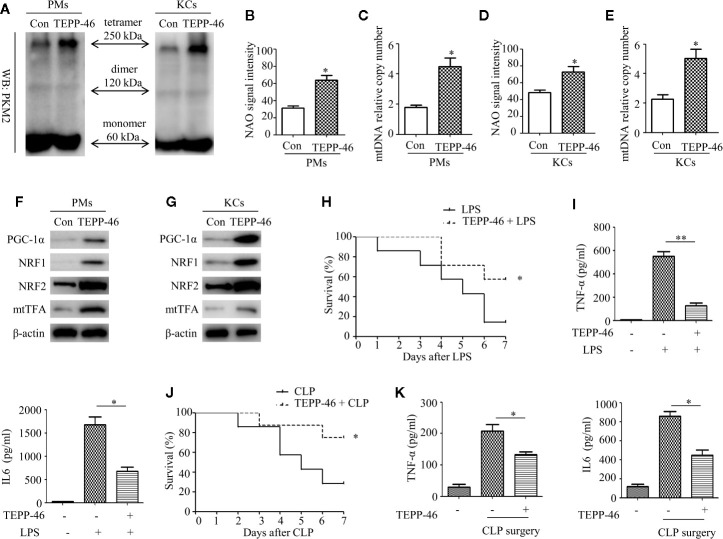
TEPP-46-mediated formation of the PKM2 tetramer protects mice from endotoxemia and sepsis *in vivo*. **(A)** Western blot analysis of PKM2 in PMs and KCs isolated from mice with (TEPP-46)/without (Con) injections of TEPP-46 (*n*=4). **(B, D)** NAO signal intensity was used to assess the mitochondrial mass in PMs and KCs isolated from mice with (TEPP-46)/without (Con) injections of TEPP-46 (*n*=3). **(C, E)** mtDNA copy number in PMs and KCs was determined by measuring MTND1 relative to B2M using RT-PCR (*n*=3). **(F, G)** Western blot analysis of PGC-1α, NRF1, NRF2, and mtTFA in PMs and KCs isolated from mice with (TEPP-46)/without (Con) injections of TEPP-46 (*n*=3). **(H)** Mouse survival was monitored every day after constructing endotoxemia models with LPS (5 mg/kg, intraperitoneally), with or without pretreatment of TEPP-46 (*n*=7, per group). **(I)** Serum TNF-α and IL-6 levels in the mice of each group (*n*=3). **(J)** Mouse survival was monitored every day after constructing sepsis models with CLP, with or without pretreatment of TEPP-46 (*n*=7, per group). **(K)** Serum TNF-α and IL-6 levels in the mice of each group (*n*=3). *p < 0.05, **p < 0.01.

We have confirmed that TEPP-46-mediated activation of PKM2 induces endotoxin tolerance by promoting mitochondrial biogenesis *in vitro*. We then asked whether TEPP-46 could protect mice from lethal endotoxemia by inhibiting cytokine release. To clarify this question, PMs and KCs were isolated from mice 4 h after the injection of TEPP-46 (50 mg/kg, intraperitoneally). We found that the protein level of the PKM2 tetramer was significantly increased under stimulation with TEPP-46, suggesting that TEPP-46 could effectively induce the activation of PKM2 in macrophages *in vivo*, similar to the *in vitro* results (**Figure 7A**). Furthermore, mitochondrial mass, mtDNA copy number and the protein levels of PGC-1α, NRF1, NRF2 and mtTFA were significantly increased in both PMs and KCs in TEPP-46-treated mice (**Figures 7B–G**). These results indicate that TEPP-46 also induced mitochondrial biogenesis in macrophages *in vivo*. To study the effect of TEPP-46 on endotoxemia in mice, mice were pretreated with TEPP-46 (50 mg/kg, intraperitoneally) or saline for 4 h and then restimulated with or without LPS (5 mg/kg, intraperitoneally). All of these mice were observed for 7 days, and we found that compared to endotoxemia mice (only LPS treated), mice pretreated with TEPP-46 (treated with TEPP-46 + LPS) had significantly improved survival (**Figure 7H**). No mice died during the observation period in the saline-treated group (control group), which was not reflected in the survival curve. In addition, the serum levels of TNF-α and IL-6 in TEPP-46-pretreated mice were significantly decreased compared to those in endotoxemia mice (**Figure 7I**). These findings suggest that TEPP-46 protects mice from LPS-induced endotoxemia by reducing the release of TNF-α and IL-6. Although LPS stimulation is a commonly used method to establish an endotoxemia model in mice, a more clinically relevant experimental model is sepsis model with a bacterial infection induced by CLP (38). To study the effect of TEPP-46 on sepsis in mice induced by CLP, the mice were divided into three groups: sham operation group, CLP group and TEPP-46 pretreatment group (TEPP-46 + CLP). All of these mice were observed for 7 days, and no mice died in the sham operation group (data not shown). The survival of mice significantly improved in TEPP-46-pretreated mice compared to in CLP mice (**Figure 7J**). As predicted, serum levels of TNF-α and IL-6 in the TEPP-46 pretreatment group were significantly decreased compared to those in the CLP group (**Figure 7K**). Collectively, these data suggest that the PKM2 tetramer agonist TEPP-46 protects mice from endotoxemia and sepsis induced by LPS or CLP by reducing the release of TNF-α and IL-6.

## Discussion

Macrophage endotoxin tolerance is defined as a hyporesponsive state in response to a secondary lethal dose of LPS following primary low-dose LPS exposure (6, 7). Studies have reported that endotoxin tolerance provides a protective mechanism to reduce the over-release of proinflammatory cytokines in response to severe infection (8, 9). In fact, endotoxin tolerance is a double-edged sword in regulating the immune response (10). In the acute inflammatory reaction stage, endotoxin tolerance serves as an important regulatory mechanism to prevent tissue damage from overactive inflammatory responses, which can cause sepsis syndrome (54). However, prolonged immune tolerance allows for the development of secondary infections, increasing mortality from sepsis, especially for immunocompromised individuals (12, 13). Therefore, further study on the mechanism of endotoxin tolerance is of great significance to better grasp the time frame of inducing immune tolerance and avoiding immunosuppression

**Figure 8:**
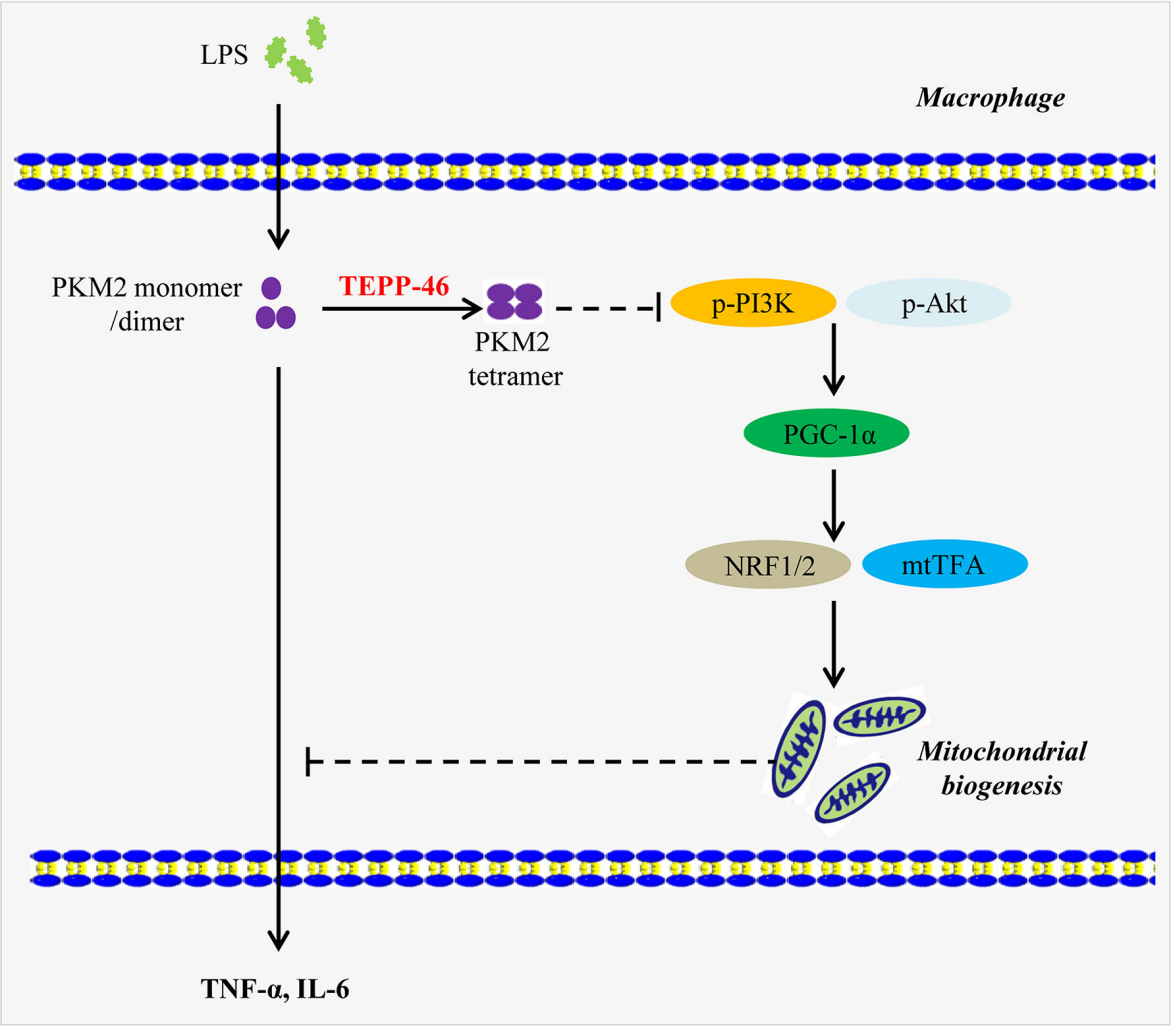
A model depicting the mechanism of macrophage tolerance to endotoxin induced by TEPP-46. The solid arrow indicates a promoting effect. The dashed line without arrow indicates an inhibiting effect. Abbreviations: lipopolysaccharide (LPS); pyruvate kinase M2 (PKM2); phosphorylated Akt (p-Akt); phosphorylated phosphatidylinositol-3-kinase (p-PI3K); peroxisome proliferator-activated receptor gamma coactivator 1 alpha (PGC-1α); nuclear respiratory factor 1/2 (NRF1/2); mitochondrial transcription factor A (mtTFA); tumor necrosis factor-α (TNF-α); interleukin 6 (IL-6).

Here, we present a novel function of the PKM2 small-molecule agonist TEPP-46 that contributes to macrophage tolerance to endotoxins by promoting mitochondrial biogenesis. As summarized in **Figure 8**, TEPP-46 induces the formation of PKM2 tetramer. Tetrameric PKM2 can promote the expression of PGC-1α by inhibiting the phosphorylation of PI3K and AKT, and promote mitochondrial biogenesis by further activating NRF1/2 and mtTFA, thus inhibiting the release of inflammatory factors mediated by LPS and promoting macrophage tolerance to endotoxins. PKM2 tetramer-induced expression of PGC-1α and PGC-1α-mediated mitochondrial biogenesis are two prerequisites for TEPP-46-mediated macrophage tolerance to endotoxins. To our knowledge, this finding is the first example of negative regulation of an LPS-mediated inflammatory response through targeting PKM2-associated mitochondrial biogenesis. This study also reveals that PGC-1α is regulated by the PI3K/Akt signaling pathway, not by the AMPK/SIRT1 signaling pathway, and plays an important role in TEPP-46-mediated macrophage tolerance to endotoxins.

PKM2 is a key enzyme of glycolysis, and its function is mainly determined by its conformation, including monomer, dimer, and tetramer (27, 28). In tumor cells and inflammatory cells, there is the transformation from a PKM2 tetramer to a PKM2 monomer/dimer (27, 28). TEPP-46 and DASA-58 are two different small-molecule agonists of PKM2 and both can promote the formation of the PKM2 tetramer, which can not only inhibit the growth of tumor cells but also inhibit the inflammatory response of immune cells by promoting the formation of the PKM2 tetramer (29–31). In the latest research, Le et al. found that PKM2 activator TEPP-46 attenuates β-aminopropionitrile fumarate (BAPN)-induced mouse model of thoracic aortic aneurysm and dissection (TAAD) by inhibiting NLRP3 inflammasome-mediated IL-1β secretion. This provides a new treatment strategy for TAAD (55). In this study, we have shown direct evidence of PKM2 tetramer-mediated macrophage endotoxin tolerance in primary macrophages (PMs and KCs), as well as in the RAW264.7 cell line. We observed increased levels of PKM2 tetramers in PMs and KCs isolated from tolerant and nontolerant mice. These findings were consistent with the *in vitro* results. In addition, we observed decreased levels of TNF-α and IL-6 after treatment with LPS in RAW264.7 cells that had been pretreated with TEPP-46.

Our next focus was to identify a mechanism by which TEPP-46 induced endotoxin tolerance in macrophages. Previously, it has been shown that TEPP-46-mediated PKM2 activation may inhibit mitochondrial dysfunction of podocytes induced by high glucose (34). DASA-58-mediated PKM2 activation suppresses osteogenesis and facilitates adipogenesis of bone marrow mesenchymal stem cells (BMSCs) by regulating β-catenin signaling and mitochondrial fusion and fission (56). In addition, a recent study reported that PKM2 is a key regulator of mitochondrial fusion to promote mitochondrial fusion and oxidative phosphorylation (OXPHOS), further modulating cancer cell growth by attenuating glycolysis (57). Obviously, these data show that activation of PKM2 is closely related to the regulation of mitochondrial function. However, the effect of PKM2 activation on the macrophage mitochondrial function is still unclear. Mitophagy and mitochondrial biogenesis maintain the relative balance of mitochondrial mass, which plays an important role in maintaining the normal function of cells (19–21). We observed a significantly increased level of mitochondrial mass in RAW264.7 cells after stimulation with TEPP-46. Decreased mitophagy or increased mitochondrial biogenesis can lead to an increase in the mitochondrial mass. Intriguingly, we did not observe changes in the protein levels of the autophagy markers LC3 and p62 in RAW264.7 cells stimulated with TEPP-46. However, we observed a significant increase in the mtDNA copy number in RAW264.7 cells stimulated with TEPP-46, which was significantly consistent with the increased protein level of mtTFA, a key regulator of mitochondrial biogenesis bound to mtDNA (22). This effect of TEPP-46 was eliminated in *PKM2*-knockdown cells, suggesting that the effect of TEPP-46 is dependent on PKM2. mtTFA is a key regulator of mitochondrial biogenesis (23). Several recent studies have reported that mtTFA upregulation augmented mitochondrial biogenesis and enhanced mitochondrial functions (22, 58). In our study, we knocked down mtTFA gene expression in RAW264.7 cells with shmtTFA lentiviruses, and this method effectively inhibited mitochondrial biogenesis. We found that silencing mtTFA inhibited RAW264.7 cell endotoxin tolerance induced by PKM2 activation, which was reflected in the levels of the inflammatory cytokines TNF-α and IL-6 in the supernatant of the mtTFA knockdown group (shmtTFA + TEPP-46 + LPS), which were increased by 85% and 111%, respectively, compared to those of the endotoxin tolerance group (TEPP-46 + LPS). In our previous study, we found that endotoxin tolerance can inhibit LPS-mediated M1-type polarization of macrophages and promote M2-type polarization (45). Our results further support the role of TEPP-46 in the negative regulation of the inflammatory response in macrophages. We found that pretreatment with TEPP-46 inhibited LPS-mediated M1-type polarization and promoted the expression of Arg1 and CD206 (M2 polarization markers). However, TEPP-46 failed to promote the expression of Arg1 and CD206 after silencing of mtTFA, suggesting that the effect of TEPP-46 is dependent on mtTFA (mitochondrial biogenesis).

PGC-1α, NRF1, NRF2, and mtTFA are the key regulatory factors that promote mitochondrial biogenesis, and the AMPK/SIRT1/PGC-1α signaling pathway is the classical pathway that induces mitochondrial biogenesis (25, 26). Therefore, we asked whether pretreatment with TEPP-46 could activate the AMPK/SIRT1/PGC-1α signaling pathway. However, we did not observe changes in the protein levels of SIRT1 and phosphorylated AMPK, suggesting that TEPP-46-promoted expression of PGC-1α was not dependent on activation of the AMPK/SIRT1 pathway. Inducing the formation of the PKM2 monomer/dimer could promote the invasion and migration of tumor cells by activating the PI3K/Akt signaling pathway (50). PI3K/Akt is the negative regulatory pathway of PGC-1α, which is involved in the regulation of various tumors and inflammatory diseases (50, 51). These results indicate that PKM2 may regulate the expression of PGC-1α by regulating the PI3K/Akt signaling pathway. Interestingly, we found that the PI3K/Akt signaling pathway was significantly suppressed in RAW264.7 cells stimulated with TEPP-46. Furthermore, pretreatment with SC79 (agonist of Akt) or 740 Y-P (agonist of PI3K) inhibited the mtDNA copy number and the protein levels of PGC-1α, NRF1, NRF2 and mtTFA induced by TEPP-46. These data indicate that activation of PKM2 induced by TEPP-46 promotes PGC-1α expression by inhibiting the PI3K/Akt signaling pathway.

Although LPS-mediated endotoxemia is a useful model for investigating sepsis, a more clinically relevant experimental model for sepsis is a bacterial infection model induced by CLP (38). In this study, we established mouse endotoxemia models and sepsis models by intraperitoneal injections of LPS and CLP, respectively. We found that the PKM2 tetramer agonist TEPP-46 protects mice from endotoxemia and sepsis induced by LPS or CLP by reducing the release of TNF-α and IL-6. By isolating mouse PMs and KCs, we found that TEPP-46 also promotes the formation of the PKM2 tetramer and induces the expression of PGC-1α, NRF1, NRF2 and mtTFA in the cell populations *in vivo* as well as *in vitro*. Collectively, these data reveal a novel pathway of TEPP-46-mediated activation of PKM2 that contributes to endotoxin tolerance by promoting mitochondrial biogenesis *in vivo* and *in vitro*.

## Data Availability Statement

The datasets presented in this study can be found in online repositories. The names of the repository/repositories and accession number(s) can be found in the article/supplementary material.

## Ethics Statement

The animal study was reviewed and approved by The Research Ethics Committee of Chongqing Medical University (No. 2017-36).

## Author Contributions

ZY and YW put forward the research hypothesis and designed the experiments. ZY, YW, WZ, and TW carried out the experimental procedures. JG carried out data analysis. YC and CM were presided the study and provided financial support. This manuscript was prepared by ZY, YW, YC, and MC. All authors contributed to the article and approved the submitted version.

## Funding

This study was supported by the National Natural Science Foundation of China (No. 81701957 and No. 81701950), China Postdoctoral Science Foundation (2019M653352) and the Kuanren Talents Program of the second affiliated hospital of Chongqing Medical University.

## Conflict of Interest

The authors declare that the research was conducted in the absence of any commercial or financial relationships that could be construed as a potential conflict of interest.
